# Characterizing enteric pathology in MPS IIIA mice suggests disease-specific vulnerability among lysosomal storage disorders

**DOI:** 10.1172/jci.insight.202780

**Published:** 2026-07-23

**Authors:** Ewa A. Ziółkowska, Letitia Williams, Elizabeth Eultgen, Grace R. Kick, Xukai Ding, Alexander Sorensen, Steven Le, Balraj Doray, Patricia I. Dickson, Robert O. Heuckeroth, Jonathan D. Cooper

**Affiliations:** 1 Department of Animal Biotechnology and Genetics, Faculty of Animal Breeding and Biology, Bydgoszcz University of Science and Technology, Bydgoszcz, Poland.; 2 Department of Pediatrics, Washington University in St. Louis, School of Medicine, St. Louis, Missouri, USA.; 3 Children’s Hospital of Philadelphia Research Institute and Perelman School of Medicine at the University of Pennsylvania, Philadelphia, Pennsylvania, USA.; 4 Department of Genetics and; 5 Department of Neurology, Washington University in St. Louis, School of Medicine, St. Louis, Missouri, USA.

**Keywords:** Gastroenterology, Genetics, Neuroscience, Neurodegeneration

## Abstract

The gastrointestinal symptoms that impair quality of life in childhood neurodegenerative disorders, may be because bowel enteric neurons are also affected.

**To the Editor:** Lysosomal storage diseases (LSDs) are fatal inherited single gene defects that impair lysosomal function (1), mostly due to deficiencies in enzymes degrading macromolecules. Lysosomal dysfunction leads to accumulation of undegraded substrates and progressive cellular dysfunction affecting many organs (1). In neuronopathic LSDs, these effects are most prominent in the central nervous system (CNS), but the rest of the body is invariably affected (1). As in other pediatric neurodegenerative disorders, affected children anecdotally have a range of gastrointestinal (GI) symptoms. We hypothesized these may be due to damage to the enteric nervous system (ENS), the complex network of neurons and glia that regulates GI motility, and other aspects of bowel function (2). Our studies in one group of neuronopathic LSDs, the neuronal ceroid lipofuscinoses (NCLs) (3), revealed progressive ENS degeneration in 3 mouse models with loss of ~50% of enteric neurons, intestinal distension, and reduced bowel motility by disease endstage (4). These effects of NCL disease upon the bowel are life limiting but can be prevented by systemic gene therapy. Human NCL bowel at autopsy also suggested profound ENS degeneration (4).

These findings suggested ENS pathology may be a general feature of neuronopathic LSDs. To test this hypothesis, we examined the ENS in endstage mice modeling Sanfillipo disease (MPS III), the form of mucopolysaccharidosis (MPS) with most pronounced CNS degeneration. MPS is a disorder of glycosaminoglycan catabolism (1), one of > 70 LSDs, each caused by a different gene defect (1). GI symptoms are pronounced in MPS III, causing significant challenges for affected children and their families (5). MPS type IIIA (MPS IIIA, Sanfillipo type A) is caused by N-sulfoglucosamine sulfohydrolase (SGSH) deficiency. *Sgsh^–/–^* mice replicate the neurological symptoms of MPS IIIA, including progressive motor decline, behavioral changes, and shortened lifespan.

We first compared levels of SGSH enzyme activity in brain and bowel of WT mice, finding 3–6 times higher activity in different bowel regions versus brain (Figure 1A), suggesting SGSH deficiency may affect bowel more than brain. Limited bowel pathology and delayed gastric emptying is evident in disease midstage *Sgsh^–/–^* mice (9.5 months) (6). In this study, we examined more severely affected *Sgsh^–/–^* mice of both sexes at disease endstage (12 months) comparing them to age-matched WT littermates and determined the extent of bowel dysmotility, enteric neuron loss, and other bowel pathologies observed in the NCLs (4). We found no overall difference in bowel length between genotypes (Figure 1B). Villus height, width, surface area, crypt depth, villus/crypt ratio, and tunica muscularis thickness in jejunum were also similar between genotypes (Figure 1, C–E), unlike the abnormalities found in NCL mice (4). Immunostaining found no evidence of overt bowel inflammation in *Sgsh^–/–^* mice (Supplemental Figure 1) (Supplemental Material; supplemental material available online with this article; https://doi.org/10.1172/jci.insight.202780DS1).

To examine the ENS, we immunostained wholemount preparations of duodenum, ileum, and colon for HuC/D (enteric neuron marker) (4). Myenteric plexus organization and neuron morphology appeared overtly normal in *Sgsh^–/–^* mice (Figure 1F), unlike the pronounced patches of enteric neuron loss evident in NCL mice (4). Neuronal density was only significantly reduced in ileum (19.3% loss versus WT, *P* = 0.0252), but not in duodenum (12.4% loss, *P* = 0.1721) and colon (24.3% loss, *P* = 0.1169) (Figure 1G). There was also a small but significant reduction in density of S100B^+^ enteric glia in the ileum but not of GFAP^+^ enteric glia (Figure 1H). These results suggest SGSH deficiency only modestly reduces myenteric plexus neuron and glial density, with no profound disruption of ENS architecture.

Intestinal motility was assessed using transit assays (4). Total bowel transit time assessed by passage of gavaged Carmine red was unaffected (Figure 1I). Segmental distribution of gavaged FITC-dextran along the bowel 2 hours after gavage was also largely normal in *Sgsh^–/–^* mice (Figure 1J). Bowel motility remains unaffected in these mice at disease endstage, despite some enteric neuron and glial loss. In contrast, NCL mice display delayed transit, fecal retention, and bowel distension correlating with extensive ENS degeneration and smooth muscle atrophy (4).

The level of SGSH activity is higher in bowel versus brain, yet our data reveal only moderate effects on enteric neurons or glia, and no overt bowel inflammation. The causes of GI symptoms in children with MPS IIIA remain unclear but likely differ from the pronounced ENS degeneration and bowel pathologies in the NCLs. The NCLs also display considerably more CNS neurodegeneration than any MPS (1, 3), suggesting that the extent of ENS degeneration may be proportional to CNS neuron loss in LSDs. Lysosomal dysfunction does not appear to affect all neurons equally, and lysosomal enzyme deficiencies may differentially affect brain versus bowel.

Our findings refine current understanding of LSD peripheral pathology, showing that severe ENS involvement is not universal but is disease specific. Each LSD should be evaluated individually to understand the full spectrum of systemic effects. These findings have important implications for treatment, with strategies that target both brain and bowel required in NCLs (4), while other approaches will need to be devised for MPS IIIA. Future studies should examine why certain lysosomal enzymes are crucial for enteric neuron survival and whether other mucopolysaccharidoses display ENS phenotypes. These studies emphasize the importance of defining disease manifestations in CNS and peripheral tissues for implementation of targeted therapies to treat these complex multisystem disorders.

## Funding support

This work is the result of NIH funding, in whole or in part, and is subject to the NIH Public Access Policy. Through acceptance of this federal funding, the NIH has been given a right to make the work publicly available in PubMed Central.

- Department of Pediatrics, Washington University in St. Louis to JDC
- NINDS National Institutes of Health grants R21 NS116574 and R01 NS140682 to JDC
- Irma and Norman Braman Endowment to ROH
- Suzi and Scott Lustgarten Center Endowment to ROH
- The Children’s Hospital of Philadelphia Frontier Program Center for Precision Diagnosis and Therapy for Pediatric Motility Disorders and Frontier Innovative Digital Pathology Imaging Seed funding to ROH
- R01DK129691 and R01DK141691 to ROH

## Conflict of interest

JDC previously received research support from BioMarin Pharmaceutical, Abeona Therapeutics, REGENXBIO, and Neurogene and is a consultant for JCR Pharmaceuticals. ROH was a consultant for BlueRock Therapeutics, and served on Scientific Advisory Board for Takeda, Neurenati Therapeutics, and the World Visceral Myopathy Foundation.

## Supplementary Material

Supplemental data

Supporting data values

## Figures and Tables

**Figure 1:**
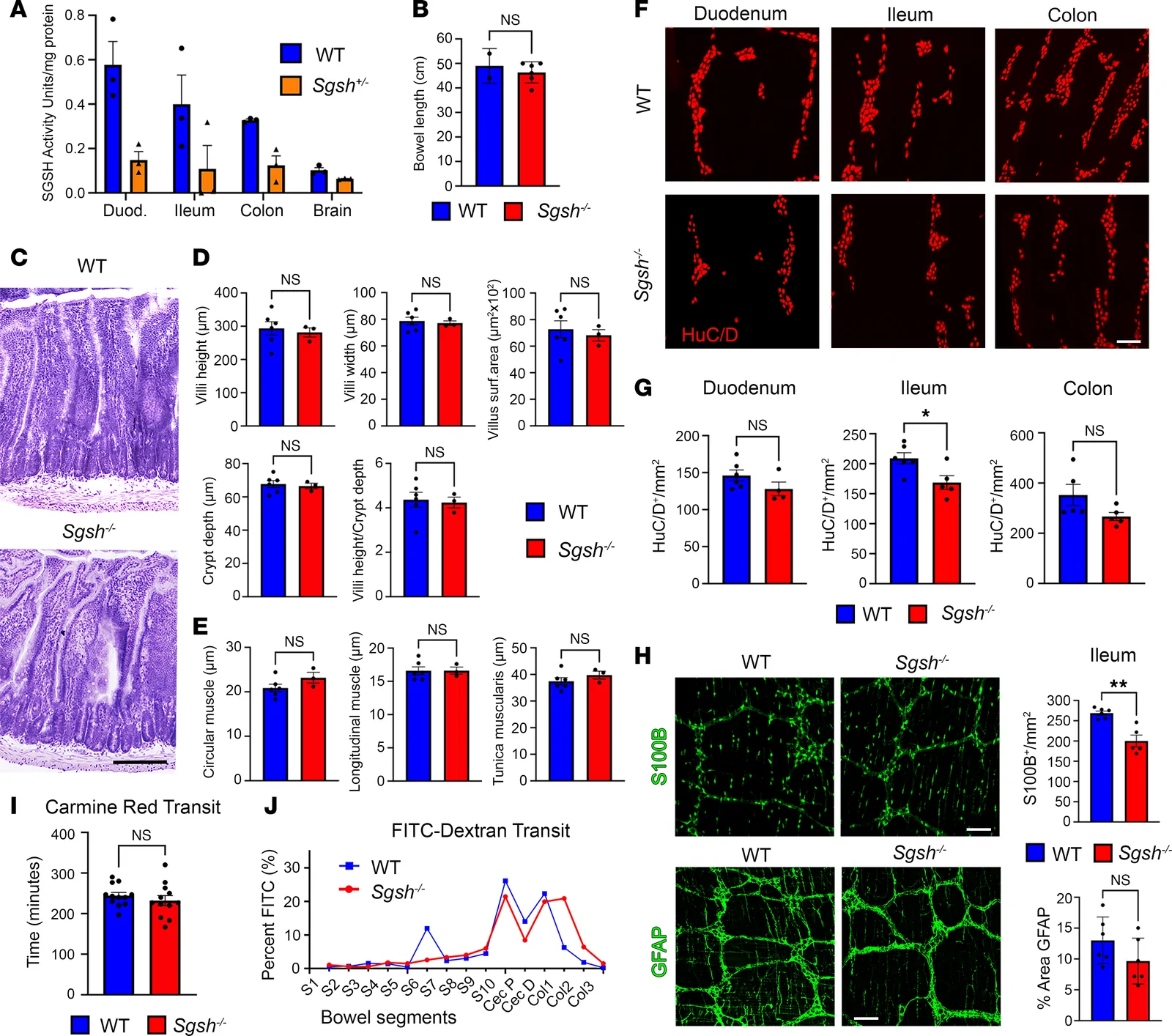
Lack of severe enteric phenotypes in disease endstage MPS IIIA mice. (**A**) SGSH enzyme activity in WT mice is higher in all bowel regions versus brain. (**B**) No significant difference in overall bowel length (pylorus to anus) between genotypes. (**C**–**E**) H&E-stained jejunal sections show normal bowel architecture with intact villi, crypts, and smooth muscle in both genotypes, with no significant difference when quantified. (**F**) HuC/D-immunostained myenteric plexus reveals well-preserved ganglion organization. (**G**) HuC/D^+^ neuron densities were statistically equivalent in duodenum and colon, with a small but significant reduction in ileum neuron density in *Sgsh^–/–^* mice. (**H**) Ileum of *Sgsh^–/–^* mice also shows significantly fewer S100B^+^ but not GFAP^+^ enteric glia. (**I** and **J**) Carmine red (*n* = 12 mice/group) and FITC–dextran assays (*n* = 13 mice/group) revealed similar whole bowel transit and segmental distribution. Scale bars: 200 μm (**C**, **F**, and **H**). Data are shown as mean ± SEM (**B**, **D**, **E**, and **G**–**I**), using unpaired 2-tailed *t* test. *P < 0.05, **P < 0.01.

## Version 1 07/23/2026

In-Press Preview

## Version 2 09/08/2026

Electronic publication
