# Supplementary material for: Characterizing enteric pathology in MPS IIIA mice suggests disease-specific vulnerability among lysosomal storage disorders
Source: JCI Insight. 2026 Jul 23;11(17):e202780. doi: 10.1172/jci.insight.202780 (PMC13564091; doi:10.1172/jci.insight.202780)
Supplement: Supplementary file 1 [file jciinsight-11-202780-s002.pdf]

Supplemental Methods

Sex as a biological variable

Our study examined male and female animals, and similar findings are reported for both sexes.

Mice

C57Bl/6J congenic wildtype (WT) and *Sgsh*$^{-/-}$ mice[1,2] (a kind gift of Dr. Haiyan Fu, UNC Chapel Hill, Chapel Hill, NC) were used in this study. These mice were maintained at Washington University School of Medicine until they reached normal disease endstage (12 months). During this time mice were provided a standard mouse chow (Purina Rodent Diet 5053) and water *ad libitum* under a 12hr light/dark cycle. Numbers analyzed are detailed in Figures. All studies used littermates and equal numbers of males and females. These studies follow ARRIVE guidelines and were performed under protocol 24-0232 approved by the Institutional Animal Care and Use Committee (IACUC) at Washington University School of Medicine in St. Louis, MO.

Bowel transit studies

Whole bowel transit: Mice fasted overnight were gavage-fed with 6% carmine red dye (300 µL) Sigma-Aldrich) in distilled water containing 0.5% methylcellulose (MilliporeSigma) and placed in individual cages with white paper covering cage bottoms[3,4]. Passage of red stool was evaluated at 10 min intervals. Each mouse was tested three times with ~3 days between tests, as previously described[3,4].

FITC-dextran transit: Mice fasted overnight were gavage-fed with 100 µL FITC-Dextran (10 mg/mL, 70,000 MW; MilliporeSigma) in distilled water containing 2% methylcellulose (MilliporeSigma)[3,4]. 2 hours after gavage, bowel from isoflurane-euthanized mice was divided into 15 segments (small intestine 1–10, proximal and distal cecum, colon 1–3). Segment contents were suspended in 100 µL 1X phosphate buffered saline, vortexed 15 s, and centrifuged (4000 rpm, 10 min). Supernatant fluorescence was measured (excitation 485 nm,

emission at 525 nm) on a plate reader. Weighted geometric mean (sum of (segment number × FITC fluorescence in that segment)/total FITC fluorescence) was calculated as described[3,4].

Wholemount bowel histology in mice

Mice were anesthetized (2% isoflurane) and transcardially perfused with PBS and decapitated. Overall bowel length was measured from pylorus to anus. The first and last 8 cm of small intestine and entire colon were cut into 2 cm lengths in cold 50 mM Tris buffered saline (TBS, pH=7.6), opened along mesenteric border, pinned to Sylgard™ 184 Silicone Elastomer (Dow Corning) using stainless steel insect pins, and fixed in fresh 4% paraformaldehyde (35 minutes colon, 45 minutes ileum, 1 hour duodenum). Fixed bowel tissue was cut into 1 cm lengths and stored in TBS/0.1% sodium azide (4°C) before staining for neuronal or glial markers[3,4]. Muscle layers were separated from mucosa and submucosa and blocked (1 hour) in TBS containing 4% Triton X-100 (TBST), 15% normal goat serum (NGS) (Jackson Immuno-Research Laboratories), then incubated in primary antibody (Supplemental Table 1) at 4°C overnight, washed (3x, TBS), and incubated in secondary antibodies (Supplemental Table 1) in 10% goat serum, 4% TBST for 2 hours[3,4]. Bowel wholemounts were mounted on Colorfrost plus slides (Thermo Fisher Scientific), dried briefly before incubation in 1x solution TrueBlack lipofuscin autofluorescence quencher (Biotium) in 70% ethanol (5 min) and rinsing (1xTBS). Slides were coverslipped in Fluoromount-G mounting medium containing DAPI (Southern Biotech).

Quantitative analysis of immunostained mouse bowel

For each sample, 10 systematically sampled, regularly spaced 20× fields (each 0.42250 mm^2) per sample were analyzed. Multi-channel images were collected on a Zeiss AxioImager Z1 microscope using StereoInvestigator (MBF Bioscience) software and exported to ImageJ (NIH). Myenteric neurons (HuC/D+ cells) and glia (S100B+ cells) were manually counted in each field

and averaged to determine cells/mm[3,4]. Because individual GFAP+ enteric glia cannot be distinguished from one another, thresholding image analysis was used to determine the percent area of each field occupied by GFAP immunoreactivity[4]. All analyses were made blind to genotype.

SGSH enzyme activity assays

SGSH (heparan-N-sulfatase) activity was determined by hydrolysis of fluorogenic substrate, 4-Methylumbelliferyl 2-deoxy-2-sulfamino-a-D-glucopyranoside following procedures from Karpova et al.[5] with some minor modifications. Briefly, 10 µl tissue homogenates were added to 20 µl 5 mM 4-MU-αGlcNS and incubated for 17h at 37°C. This first incubation was stopped with 6 µl of McIlvaine's 2X phosphate/citrate buffer pH 6.7 and then the second step started by adding 10 µl (0.1U) α-glucosidase and incubated for another 24hr at 37°C. 200 µl of glycine carbonate to stop this second reaction. 200 µl of each sample was transferred to a 96 well plate and fluorescence read on a Biotek - Synergy H1 microplate reader Ex 365 nm, Em 440 nm. A unit of SGSH activity was defined as release of 1 nmol of 4-methylumelliferone (4MU) per hour. Protein concentration was estimated by the Bradford method, using BSA as standard.

Bowel histology and Analyses

Immediately after decapitation, jejunum was harvested and fixed overnight at 4°C in fresh 4% paraformaldehyde. Fixed pieces of jejunum were embedded in paraffin blocks and cross sectioned at 10 µm thickness using a sledge microtome. Sections were deparaffinized and stained with hematoxylin and eosin, or immunostained with markers of bowel macrophages (Iba1, CD68), leukocyte common antigen CD45, helper (CD4) or cytotoxic T cells (CD8) (antibodies listed in Supplemental Table 1). Then, measurements were made of the height and width of the intestinal villi, villi surface area, the depth of the crypts, ratio of villi height to crypt depth, and the thickness of both circular and longitudinal smooth muscular layers individually

and their combined thickness in at least 5 sections per individual, with 50 measurements made in each section[4].

Statistical analyses

Statistical analyses of bowel and CNS histology were performed using GraphPad Prism version 9.1.0 for MacOS (GraphPad Software, San Diego, CA). Unpaired t-tests were used for comparisons between two groups. A p-value of ≤0.05 was considered significant.

Supplemental Table 1 Antibodies used

Antibody	Concentration	Catalog number	Source
Rabbit anti (HuC/D)	1:500 (mouse bowel)	ab184267	Abcam<br>RRID: AB_2864321
Rabbit anti-GFAP	1:10000 (mouse bowel)	Z0334	Agilent<br>RRID: AB_10013382
Rabbit anti-S100 beta	1:300 (mouse bowel)	ab52642	Abcam<br>RRID: AB_882426
Rat anti-CD68	1:400 (mouse brain)	MCA1957	Biorad<br>RRID: AB_322219
Rabbit anti-Iba1	1:2000 (mouse bowel)	019-19741	FUJIFILM Wako<br>RRID: AB_839504
Rat anti-CD4	1:100 (mouse bowel)	MCA1767	Biorad<br>RRID:AB_322769
Rat anti-CD8	1:100 (mouse bowel)	MCA609G	Biorad<br>RRID: AB_321407
Rat anti-CD45	1:100 (mouse bowel)	MCA1031G	Biorad<br>RRID:AB_321730
AlexaFluor goat anti-rat 488	1:400 (mouse bowel)	A-11006	ThermoFisher Scientific (Invitrogen)<br>RRID:AB_2534074
AlexaFluor goat anti-rabbit 488	1:400 (mouse bowel)	A-11008	ThermoFisher Scientific (Invitrogen)<br>RRID: AB_143165
Alexa Fluor goat anti-rabbit 546	1:400 (mouse bowel)	A-11035	ThermoFisher Scientific (Invitrogen)<br>RRID: AB_2534093

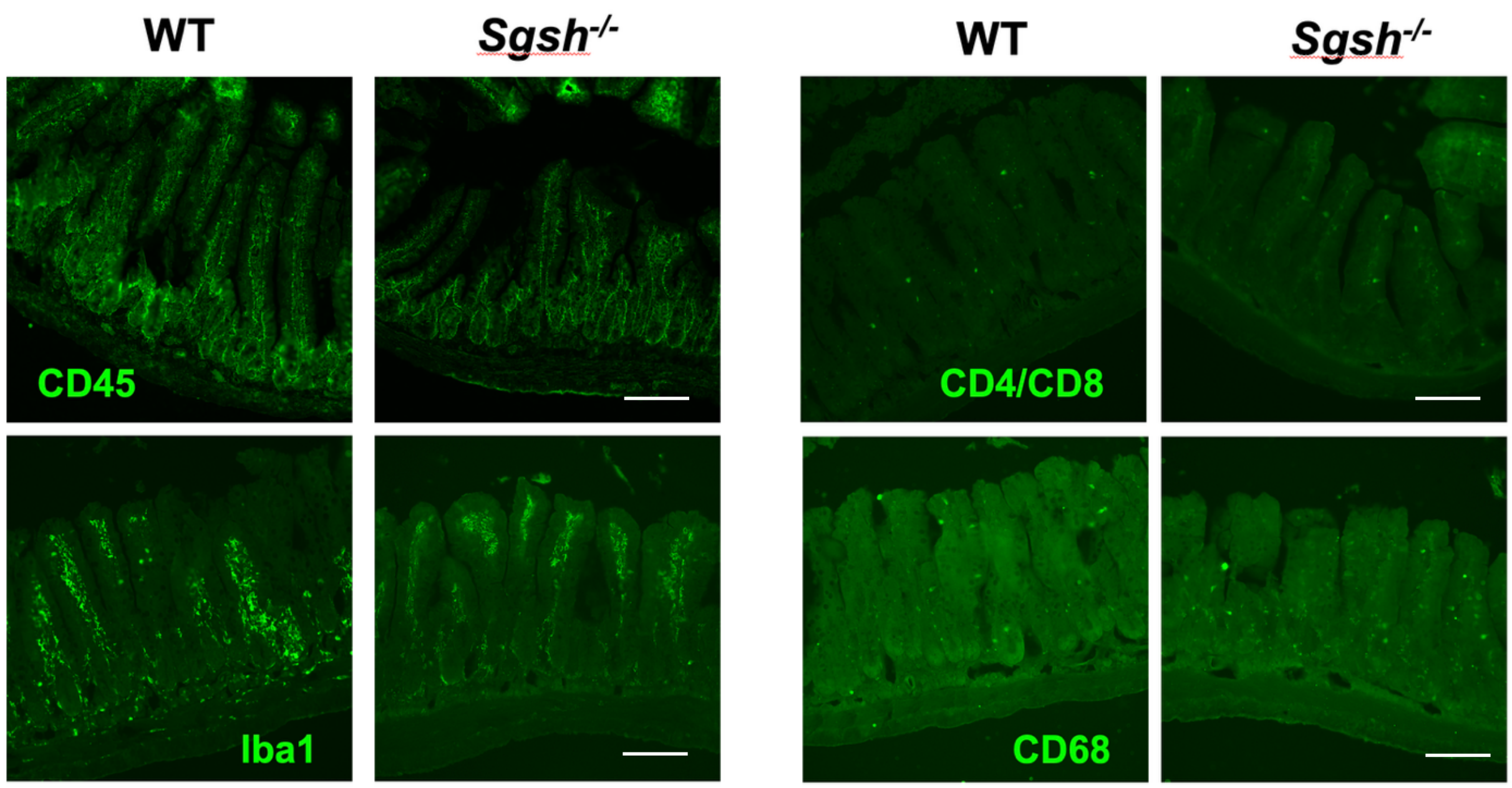

Supplemental Figure 1

Immunostaining for bowel macrophages (Iba1, CD68), leukocyte common antigen CD45, and simultaneous staining for helper (CD4) or cytotoxic T cells (CD8) reveals no obvious increase in any marker in the jejenum of disease endstage *Sgsh*$^{-/-}$ mice compared to age-matched wildtype (WT) mice. Scale bar = 100µm.

Data availability: The raw data used and/or analyzed during the current study are in the supporting data file with detail on experimental design and procedures contained in the Supplemental Material file.

Acknowledgements: The MPS IIIA mice were kindly supplied by Dr. Haiyan Fu, UNC Chapel Hill. We thank Dr. Alison Barnwell for constructive comments on the manuscript.

Authors' contributions: JDC, PID, ROH and EAZ conceived and designed the study; JDC, ROH, obtained funding; EAZ, JDC carried out the bowel preparations; EAZ, GRK and XD performed mouse bowel immunostaining and quantitative analyses; EAZ prepared all the figures with JDC; LLW performed bowel transit studies; AS and SQL managed the mouse colonies, generated the mice for these studies and performed all genotyping with the assistance of BD and EME; SQL and BD performed the SGSH enzyme activity assays; EAZ performed bowel histology, immunostaining and analyses of villi, crypts, bowel smooth muscle and bowel length; JDC, PID and ROH supervised all studies; EAZ, PID, JDC and ROH interpreted data; EAZ and JDC wrote the manuscript with input from all the authors. All authors read and approved the final manuscript.

References cited

1. Bhaumik M, Muller VJ, Rozaklis T, Johnson L, Dobrenis K, Bhattacharyya R, Wurzelmann S, Finamore P, Hopwood JJ, Walkley SU, Stanley P. A mouse model for mucopolysaccharidosis type III A (Sanfilippo syndrome). *Glycobiology* 1999; 9(12):1389-96.

2. Fu H, Cataldi MP, Ware TA, Zaraspe K, Meadows AS, Murrey DA, McCarty DM. Functional correction of neurological and somatic disorders at later stages of disease in MPS IIIA mice by systemic scAAV9-hSGSH gene delivery. *Mol. Therapy Methods Clin. Develop*. 2016; 3:16036'

3. Ziółkowska EA, Jansen MJ, Williams LL, Wang SH, Eultgen EM, Takahashi K, Le SQ, Nelvagal HR, Sharma J, Sardiello M, DeBosch BJ, Dickson PI, Anderson JB, Sax SE, Wright CM, Bradley RP, Whiteman IT, Makita T, Grider JR, Sands MS, Heuckeroth RO, Cooper JD. Gene therapy ameliorates bowel dysmotility and enteric neuron degeneration and extends survival in lysosomal storage disorder mouse models. *Sci Transl Med.* 2025;17(781):eadj1445.

4. Ziółkowska EA, Williams LL, Jansen MJ, Wang SH, Eultgen EM, Sharma J, Sardiello M, Bradley RP, Whiteman IT, Sands MS, Heuckeroth RO, Cooper JD. Enteric nervous system degeneration in human and murine CLN3 disease, is ameliorated by gene therapy in mice. *Acta Neuropathol Commun.* 2025;13(1):260.

5. Karpova EA, Voznyi YaV, Keulemans JL, Hoogeveen AT, Winchester B, Tsvetkova IV, van Diggelen OP. A fluorimetric enzyme assay for the diagnosis of Sanfilippo disease type A (MPS IIIA). *J Inherit Metab Dis*. 1996;19(3):278-85.
